# Increasing incidence of acute autoimmune hepatitis: a nationwide survey in Japan

**DOI:** 10.1038/s41598-020-71296-0

**Published:** 2020-08-28

**Authors:** Atsushi Takahashi, Hiromasa Ohira, Kazumichi Abe, Mikio Zeniya, Masanori Abe, Teruko Arinaga-Hino, Takuji Torimura, Kaname Yoshizawa, Akinobu Takaki, Jong-Hon Kang, Yoshiyuki Suzuki, Nobuhiro Nakamoto, Ayano Inui, Atsushi Tanaka, Hajime Takikawa

**Affiliations:** 1 Department of Gastroenterology, Fukushima Medical University School of Medicine, 1 Hikarigaoka, Fukushima, 960-1295 Japan; 2 Sanno Medical Center, International University of Health and Welfare, 8-10-16 Akasaka, Minato-ku, Tokyo, 107-0052 Japan; 3 Department of Gastroenterology and Metabology, Ehime University Graduate School of Medicine, Shitsukawa, To-on, Ehime 791-0295 Japan; 4 Department of Medicine, Kurume University School of Medicine, 67 Asahi-machi, Kurume-shi, Fukuoka, 830-0011 Japan; 5 Department of Gastroenterology, National Hospital Organization, Shinshu Ueda Medical Center, 1-27-21 Midorigaoka, Ueda-City, Nagano 386-8610 Japan; 6 Department of Gastroenterology and Hepatology, Okayama University Graduate School of Medicine, Dentistry, and Pharmaceutical Sciences, 2-5-1 Shikata-cho, Kita-ku, Okayama, 700-8558 Japan; 7 Center for Gastroenterology, Teine Keijinkai Hospital, 1-12 Maeda, Teine-ku, Sapporo, 006-8555 Japan; 8 Department of Hepatology, Toranomon Hospital, 2-2-2 Toranomon, Minato-ku, Tokyo, 105-8470 Japan; 9 Department of Internal Medicine, Keio University School of Medicine, 35 Shinanomachi, Shinjuku-ku, Tokyo, 160-8582 Japan; 10 Department of Pediatric Hepatology and Gastroenterology, Saiseikai Yokohamashi Tobu Hospital, 3-6-1 Shimosueyoshi, Tsurumi-ku, Yokohama City, Kanagawa, 230-0012 Japan; 11 Department of Medicine, Teikyo University School of Medicine, 2-11-1 Kaga, Itabashi-ku, Tokyo, 173-8605 Japan; 12 Faculty of Medical Technology, Teikyo University, 2-11-1 Kaga, Itabashi-ku, Tokyo, 173-8605 Japan

**Keywords:** Gastroenterology, Hepatology, Medical research, Epidemiology

## Abstract

The Japanese diagnostic guidelines for autoimmune hepatitis (AIH) were proposed in 2014. This study aimed to determine the trends and characteristics of AIH based on a Japanese nationwide survey. Data for 796 patients who were newly diagnosed with AIH from 2014 to 2017 were collected from January to March, 2019 from 54 hospitals throughout Japan. Clinical characteristics, including treatment, were compared with those reported in a prior 2015 survey. The population had a median age of 63 years at diagnosis, and the male to female ratio was 1:5.3. The numbers of women was significantly lower in this survey than in the 2015 survey. Moreover, the incidence of AIH with histological acute hepatitis increased significantly from 11.0 to 21.7%. The changes in the laboratory findings, such as in transaminase and immunoglobulin G levels and antinuclear antibody titers, as well as in prednisolone treatment, reflected an increasing incidence of acute AIH. The clinical characteristics of AIH changed rapidly, in parallel with the increasing incidence of acute AIH. The elucidation and diagnosis of AIH with acute hepatitis are important in the management of AIH.

## Introduction

Although autoimmune hepatitis (AIH) with an acute presentation is difficult to diagnose based on a scoring system because of low immunoglobulin G (IgG) levels and antinuclear antibody (ANA) titers, its clinical and histological characteristics have been gradually elucidated by several reports^1–18^. AIH with an acute presentation includes two types: (1) the acute exacerbation phase, in which patients present with the clinical features of acute hepatitis, with histological evidence of chronic hepatitis; and (2) the acute hepatitis phase, in which patients present with histological features of acute hepatitis^11^.

Regular nationwide surveys of AIH have been conducted since 1975 in Japan^1,2,19–21^. The 2015 survey reported the trends in 1682 patients who were diagnosed with AIH from 2009 to 2013^2^. The proportion of AIH cases with acute hepatitis (11.7%) in 2015 was similar to that in 2009 (10.9%); notably, 1,056 patients were diagnosed with AIH from 2006 to 2008^1^.

The accumulation of findings of AIH with an acute presentation^1–18^ and its novel diagnostic guidelines^22^ may have affected the actual characteristics of AIH in Japan after the previous 2015 survey. The aim of this study was to elucidate the recent trends in AIH in Japan by comparing the results of the 2015 survey to a 2018 survey.

## Results

### Characteristics of patients with AIH

The survey population had a median age at diagnosis of 63 years, and the male-to-female ratio was 1:5.3. Compared with the 2015 survey, the 2018 survey had a significantly lower proportion of women (Table 1). The distribution of age at diagnosis had a single peak in the 60 s in both surveys.

**Table 1:** Characteristics of patients upon the diagnosis of autoimmune hepatitis in 2018 and 2015.

	2018		2015		2015 vs. 2018
Variable	Value	Available no. of patients	Value	Available no. of patients	*P*
Age (years)	63 (52–70)	783	62 (53–70)	1,386	0.592
Female (%)	84.0 (669)	796	88.1 (1,238)	1,405	0.009
AST (U/L)	216 (86–512)	788	187 (81–507)	1,407	0.020
ALT (U/L)	255 (91–628)	788	214 (84–543)	1,408	0.011
ALP (U/L)	430 (322–614)	781	424 (306–587)	1,393	0.304
$\gamma$-GTP (U/L)	158 (86–289)	786	146 (80–260)	1,395	0.041
Total bilirubin (mg/dL)	1.2 (0.8–4.0)	786	1.1 (0.7–2.8)	1,394	0.024
Prothrombin time (INR)	1.09 (1.02–1.21)	719	–	–	–
Prothrombin time (%)	84 (69–96)	730	–	–	–
IgG (mg/dL)	2090 (1,670–2,697)	769	2,251 (1835–2,885)	1,378	< 0.001
ANA titer	160 (40–640)	764	160 (80–640)	1,362	< 0.001
ANA positivity	86.2% (674)	782	93.8% (1,312)	1,398	< 0.001
ASMA positivity	37.4% (96)	257	42.4% (191)	450	0.213
AMA positivity	14.8% (107)	725	15.9% (107)	675	0.622
LKM1 antibody positivity	6.0% (13)	218	13.5% (56)	414	0.006
Revised IAIHSG score	15 (13–18)	731	15 (13–18)	1,409	0.412
Simplified score	6 (5–7)	693	6 (5–7)	1,410	0.051
Hepatitis B surface antigen positivity	0.6% (4)	680	0.3% (4)	1,402	0.503
Hepatitis B core antibody positivity	17.4% (105)	605	21.6% (175)	809	0.054
Hepatitis C antibody positivity	1.4% (11)	776	3.2% (45)	1,391	0.016
Hepatitis C RNA positivity	1.8% (3)	164	8.1% (24)	296	0.011
HLA-DR4 positivity	63.5% (80)	126	68.9% (182)	264	0.339
HLA-DR2 positivity	6.6% (8)	122	9.6% (24)	251	0.438
**Histologic diagnosis**					
Acute hepatitis	21.7% (157)	723	11.0% (135)	1,225	< 0.001
Chronic hepatitis	70.0% (506)	723	81.9% (1,003)	1,225	< 0.001
Liver cirrhosis	8.3% (60)	723	7.1% (87)	1,225	0.380
**Basic histology**					
Interface hepatitis 0/1/2	31/107/559	697	35/270/901	1,206	0.014
Portal inflammation 0/1/2	25/130/535	690	41/273/867	1,181	0.059
Plasma cell infiltration 0/1/2	69/178/436	683	106/437/584	1,127	< 0.001
Lobular necrosis/inflammation 0/1/2	109/187/383	679	97/410/617	1,124	0.431
Fibrosis 0/1/2/3/4	91/234/177/104/45	651	105/387/352/207/82	1,133	0.005
Frequency of bile duct injury	22.4% (147)	656	23.8% (251)	1,055	0.549
Frequency of hepatocyte rosette formation	35.9% (220)	613	43.8% (418)	955	0.002
Frequency of centrilobular necrosis	36.2% (204)	564	33.0% (251)	761	0.250
Frequency of emperipolesis	28.1% (112)	398	14.5% (68)	469	< 0.001
Frequency of fatty change	18.1% (125)	691	17.8% (212)	1,192	0.917

Values are expressed as median (25–75 percentile) for continuous variables or as the number of patients or as percentage (number) for categorical variables.

ALP, alkaline phosphatase; ALT, alanine aminotransferase; ANA, antinuclear antibody; ASMA, anti-smooth muscle antibody; AST, aspartate aminotransferase; $\gamma$-GTP, gamma-glutamyl transpeptidase; HLA, human leukocyte antigen; IAIHG, International AIH Study Group; IgG, immunoglobulin G; LKM, liver/kidney microsomal.

Of the 760 patients, 147 (19.3%) had a history of alcohol intake and 56 (38.1%) drank < 20 g of ethanol per day. Of the 780 patients, 115 (14.7%) had a history of medications associated with the development of AIH; the major kinds of drugs were calcium channel blockers (13/115, 11.3%), angiotensin II receptor blockers (12/115, 10.4%), and herbal medicines (10/115, 8.7%). Only 6 of 687 patients with AIH (0.9%) had a family history of AIH. No patients developed AIH with biological and checkpoint inhibitor therapies.

### Laboratory findings

**Figure 1:**
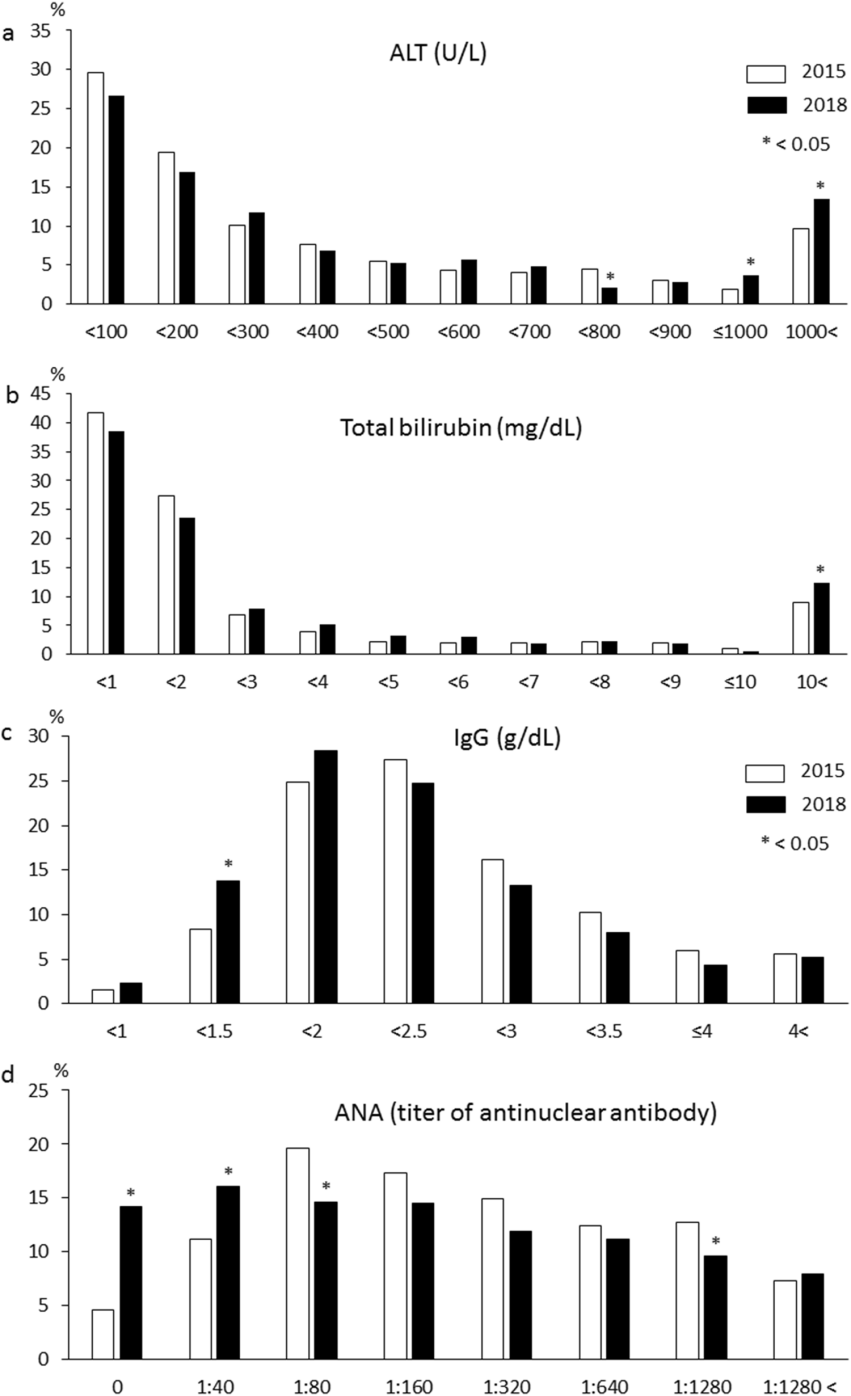
Comparison between the 2015 survey (white bar) and the 2018 survey (black bar) in the distribution of serum (**a**) alanine aminotransferase (*ALT*) levels, (**b**) total bilirubin levels, (**c**) immunoglobulin G (*IgG*) levels, and (**d**) antinuclear antibody (*ANA*) titers at diagnosis in patients with autoimmune hepatitis.

The levels of aminotransferases and total bilirubin were significantly higher in the 2018 survey than in the 2015 survey (Table 1). As shown in Fig. 1, compared with the patients in the 2015 survey, those in the 2018 survey had: significantly increased proportions of cases with alanine aminotransferase (ALT) levels > 1,000 U/L and total bilirubin > 10 mg/dL; significantly lower IgG levels, but a significantly higher proportion of cases with IgG concentration of 1–1.5 g/dL; and, of 674 cases, a significantly decreased proportion had serum ANA positivity (86.2% vs. 93.8%, *p* < 0.001) and an ANA titer of 1:80, but a significantly increased proportion had an ANA titer ≤ 1:40. Most patients positive for liver-kidney microsomal (LKM) antibody were also positive for ANA or anti-smooth muscle antibody. Only one patient (0.5%) in the 2018 survey and two patients (0.5%) in the 2015 survey were positive for LKM antibody alone; they were classified as having type 2 AIH. On the other hand, 692 patients (88.5%) in the 2018 survey and 1,357 (97.1%) in the 2015 survey were classified as having type 1 AIH. No patients had other autoantibodies, such as antibodies against soluble liver antigen/liver pancreas or antineutrophil cytoplasmic antibody.

### Liver histological findings

Of the 723 patients whose data on histological assessment of the liver were available, 70.0% were diagnosed with chronic hepatitis and 21.7% were diagnosed with acute hepatitis; the latter was significantly more frequent in the 2018 survey than in the 2015 survey (21.7% vs. 11.0%, *p* < 0.001) (Table 1). The frequency of acute hepatitis was also significantly increased, from 13.8% in 2015 to 23.7% in 2018, in the 28 facilities that completed both surveys (Supplemental Table 2). With regard to basic histological characteristics of the cases in the 2018 survey, compared with those in the 2015 survey, interface hepatitis and plasma cell infiltration were significantly more pronounced; the number of cases that had no fibrosis was higher, despite the similar frequency of centrilobular necrosis; emperipolesis was significantly increased; and hepatocyte rosette formation was decreased.

**Table 2:** Diagnosis and severity of autoimmune hepatitis.

	2018		2015		2015 vs. 2018
	Frequency/value	Available no	Frequency/value	Available no	*P*
**Diagnosis**					
*Japanese guide 2013*					
Typical	80.4% (562)	699	–		
Atypical	19.6% (137)	699	–		
*Revised score*			–		
Definite	48.8% (385)	789	48.0% (677)	1,409	0.770
Probable	47.8% (377)	789	48.3% (680)	1,409	0.864
Other	3.4% (27)	789	3.7% (52)	1,409	0.838
*Simplified score*					
Definite	45.7% (349)	763	38.2% (538)	1,410	< 0.001
Probable	31.1% (237)	763	33.6% (476)	1,410	0.219
Other	23.2% (177)	763	28.1% (396)	1,410	0.016
**Severity**					
Mild	38.5% (297)	771			
Moderate	43.6% (336)	771			
Severe	17.9% (138)	771			
*(Clinical signs)*					
Hepatic encephalopathy	1.9% (14)	753			
Reduction or disappearance of hepatic dullness	2.1% (15)	710			
*(Clinical laboratory tests)*					
AST/ALT of more than 200 U/L	60.9% (480)	788			
Bilirubin of > 5 mg/dL	21.6% (170)	786			
Prothrombin time < 60%	16.2% (119)	734			
*(Imaging tests)*					
Hepatic atrophy	4.2% (31)	745			
Heterogeneous liver parenchyma	11.1% (82)	740			

Values are given as the percentage (number).

ALT, alanine aminotransferase; AST, aspartate aminotransferase.

### Diagnosis and severity of AIH

Most patients were diagnosed within 6 months from estimated onset in both surveys (2018: 79.0%, 2015: 77.2%; *p* = 0.361). In the 2018 survey, 80.4% had typical cases, based on the Japanese guidelines; whereas definite cases were seen in 48.8%, based on the revised score, and 45.7%, based on the simplified score (Table 2). The severity of AIH was mild in 38.5%, moderate in 43.6%, and severe in 17.9%. Of the items evaluating severity, clinical laboratory tests had relatively high frequencies of abnormalities, whereas clinical signs had low frequencies of abnormalities.

### Comorbidity

Other autoimmune diseases were present in 194 of 787 patients (24.7%) with AIH, and this frequency was similar to that in the 2015 survey (24.9%) (Table 3). In the 194 AIH patients with other autoimmune diseases, the most frequent was chronic thyroiditis (8.3%), followed by Sjögren’s syndrome (6.6%), rheumatoid arthritis (2.7%), and primary biliary cholangitis (2.4%). The order by frequency was exactly the same as in the previous 2015 survey. However, the frequency of malignancy increased significantly from 6.4% in 2015 to 10.3% in the present survey (*p* = 0.001). In patients with malignancy, the most frequent complication was gastric cancer (1.8%), followed by colon cancer (1.7%) and breast cancer (1.7%).

**Table 3:** Comorbidity and Treatment in patients with autoimmune hepatitis in 2018 and 2015.

	No. of patients		2015 versus 2018
	2018	2015	*P*
**Comorbidity**			
*Autoimmune disorder*	24.7% (194/787)	24.9% (347/1,395)	0.948
Chronic thyroiditis	8.3% (65)	8.0% (111)	
Sjögren’s syndrome	6.6% (52)	6.6% (92)	
Rheumatoid arthritis	2.7% (21)	3.7% (51)	
Primary biliary cholangitis	2.4% (19)	2.8% (39)	
Systemic sclerosis	1.8% (14)	0.8% (11)	
Graves’ disease	1.8% (14)	1.1% (16)	
Systemic lupus erythematosus	1.5% (12)	2.4% (34)	
Idiopathic thrombocytopenic purpura	0.6% (5)	0.7% (10)	
Raynaud’s phenomenon	0.5% (4)	0.9% (12)	
Dermatomyositis/polymyositis	0.5% (4)	0.4% (6)	
Others	2.3% (18)	1.9% (26)	
*Malignancy*	10.3% (81/784)	6.4% (88/1,383)	0.001
Gastric cancer	1.8% (14)	0.8% (11)	
Colon cancer	1.7% (13)	0.7% (9)	
Breast cancer	1.7% (13)	1.0% (14)	
Uterine or ovarian cancer	1.0% (8)	0.4% (6)	
Lung cancer	0.9% (7)	0.5% (7)	
Hepatocellular carcinoma	0.6% (5)	0.8% (11)	
Others	2.9% (23)	2.2% (31)	
**Treatment**			
Prednisolone	85.5% (665/778)	80.9% (1,129/1,396)	0.008
Prednisolone alone	31.8% (204/642)	30.0% (331/1,103)	0.473
Initial dose of prednisolone (mg/day)	40 (30–40)	30 (30–40)	< 0.001
Maintenance dose of prednisolone (mg/day)	5 (5–7.5)	5(5–7.5)	0.281
Steroid pulse therapy, n (%)	19.1% (122/640)	13.3% (138/1,036)	0.002
Prednisolone + ursodeoxycholic acid	63.0% (409/649)	64.7% (714/1,103)	0.503
Efficacy of prednisolone	97.8% (575/588)	97.6% (1,008/1,033)	0.923
Relapse during prednisolone therapy	23.2% (141/609)	24.7% (247/1,000)	0.520
Azathioprine	12.3% (80/648)	9.4% (128/1,362)	0.051
Ursodeoxycholic acid alone	10.9% (81/744)	15.6% (213/1,362)	0.003
Without treatment	2.4% (18/744)	2.0% (27/1,363)	0.612
Biochemical remission after 6 months of therapy	58.4% (330/565)	-	-

Values are given as the percentage (number) for categorical variables or as median (25^th^–75th percentile) for continuous variables.

### Treatment

**Figure 2:**
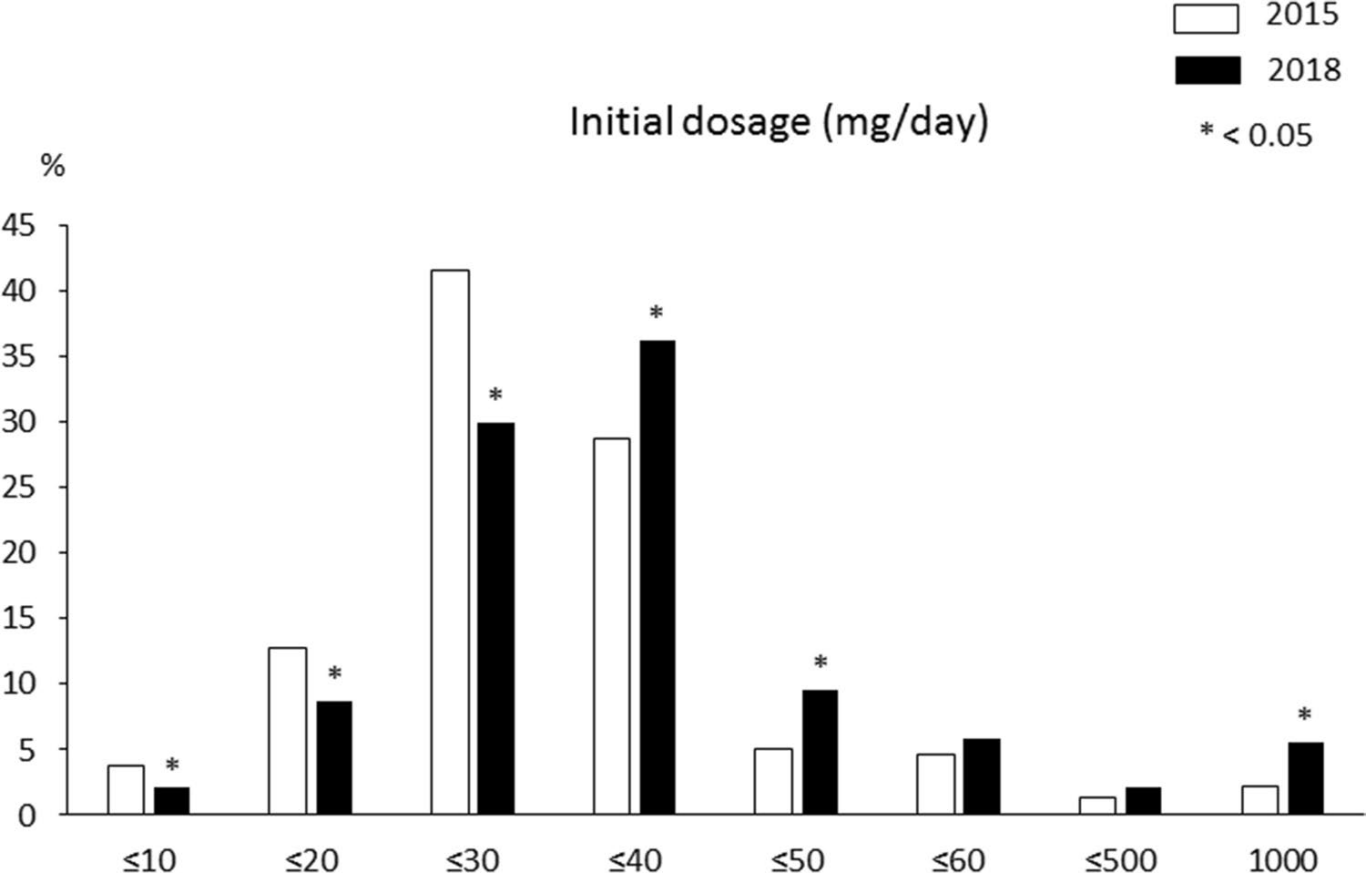
Comparison between the 2015 survey (white bar) and the 2018 survey (black bar) in the distribution of the initial dosage of prednisolone.

There were 665 of 778 patients (85.5%) who were treated with prednisolone; of these, 409 (63.0%) were treated with a combination of prednisolone and ursodeoxycholic acid (UDCA), and 204 (31.8%) were treated with prednisolone alone (Table 3). Furthermore, 122 patients (19.1%) were treated with steroid pulse therapy. Compared with the 2015 survey, the 2018 survey had significantly higher frequencies of prednisolone treatment (85.5% vs. 80.9%; *p* = 0.008), including pulse (19.1% vs. 13.3%; *p* = 0.002). The initial dose of prednisolone was higher in the 2018 survey than in the 2015 survey (40 mg vs. 30 mg, *p* < 0.001). Initial dosages of 31–50 mg daily were significantly increased, while those of ≤ 30 mg daily were significantly decreased (Fig. 2). The 2018 and 2015 surveys had similar efficacy rates of prednisolone (97.8% vs. 97.6%, respectively; *p* = 0.923) and rates of relapse during prednisolone treatment (23.2% vs. 24.7%, respectively; *p* = 0.520). Eighty patients (12.3%) were treated with azathioprine. Of the 113 patients who did not receive prednisolone treatment, 81 (71.7%) were treated with UDCA alone and 18 (15.9%) were followed up without treatment. The rate of biochemical remission after 6 months of therapy was 58.4% in the 2018 survey. The rate was significantly higher in patients receiving prednisolone alone than in those receiving UDCA alone (68.4% vs. 32.7%, respectively; *p* < 0.001).

### Outcome

During the 3-year survey period, 9 patients (1.1%) died of liver-related causes and 1 patient (0.1%) received a liver transplant. The male-to-female ratio was 1:2.3, and the mean age at diagnosis was 63.2 years in these 10 patients.

## Discussion

The prevalence of acute AIH has not been reported in countries other than Japan. The Japanese nationwide survey in 2009 was the first to report that 95 of 871 (10.9%) patients with AIH had acute hepatitis^1^; this frequency remained steady at 11.7% in the 2015 survey^2^, but it increased significantly to 21.7% in the 2018 survey. To the best of our knowledge, this evidence of an increasing trend of acute AIH was the first to be reported worldwide.

In the present survey, the high prevalence of acute AIH may have affected the AIH characteristics found. The high number of cases that showed no fibrosis on histology was likely directly reflective of the high prevalence of AIH with acute hepatitis. On the other hand, the increased incidence of acute hepatitis was reflected more in the laboratory findings than in the histological findings. In addition to the elevated levels of aspartate aminotransferase (AST), ALT, and total bilirubin, a low IgG level and a low ANA titer were found as characteristics of acute AIH in the previous survey^14,16^. The changes in prednisolone treatment, such as the initial dose or frequency of pulse therapy, may have reflected the increasing incidence of acute hepatitis. The reason could be that, in the 2015 survey of AIH patients, the initial dosage of prednisolone was higher and pulse therapy was more frequent in patients with acute hepatitis than in those with chronic hepatitis^2^.

The precise reason for the increasing incidence of acute AIH has not been elucidated, although some factors can be considered. First, the Japanese diagnosis and treatment guidelines that were proposed in 2014^22^ may have led to increased diagnosis of AIH, including acute presentation. In fact, in the present survey, the frequency of typical cases, as defined by the Japanese guidelines, was higher than that of definite cases, as defined by the revised or simplified criteria of the IAIHG. Second, in 2015 in Japan, AIH was designated an intractable disease that can be treated with free drugs. However, fulminant hepatitis was excluded from this list of designated intractable diseases. This system change may have increased the diagnosis of acute liver failure as AIH. Third, the biases of the specific hospitals and clinics that completed the present survey may have affected the results. However, the present results showed a trend of increasing incidence of AIH with acute hepatitis, regardless of the facilities.

In the present survey, there were some changes that cannot be attributed to the increasing incidence of acute hepatitis. The significant increase in the incidence of malignancy can reflect the increasing trend in the general population^23^. Moreover, the present study included relatively fewer women, although the male-to-female ratio of 1:5.3 in the present study was the same as the 1:4.3 ratio in a recent epidemiological study in Japan^24^.

Although the strengths of the present study were the large sample size and the new trend in AIH, there are several limitations. First, the number of subjects in the 2018 survey was lower than in the 2015 survey, because it was more detailed and took more time. However, the 2018 survey provided new information, such as severity and treatment responses. Furthermore, the trend of acute hepatitis was confirmed in the facilities that completed both the 2015 and 2018 surveys. The second limitation of the present study was the definition of acute hepatitis, which was based on histological findings by each pathologist. Therefore, acute hepatitis cases in the present survey may have included acute exacerbation of chronic hepatitis, because the histological definition of acute hepatitis has not been elucidated. On the other hand, previous nationwide surveys in Japan have reported the prevalence of acute hepatitis using the same definition; thus, use of the same definition of acute hepatitis would allow a comparison of its prevalence. Although the two phenotypes of acute presentation in the Japanese guidelines are not largely different from the concepts in the European Association for the Study of the Liver clinical practice guidelines^24^, validation of the definition in other cohorts, not only in Japan but also in other countries, is needed. Third, ANA is measured mostly by indirect immunofluorescence (IF) methods using human epithelial (HEp-2) cells in Japan. The high correlation between measurement using frozen liver tissue and that using HEp-2 cells is confirmed; therefore, indirect IF methods using HEp-2 cells are recommended in Japan. Fourth, patients without liver histology were included if they satisfied the revised or simplified criteria. Therefore, patients with drug-induced liver injury or drug-induced AIH-like injury may have been included in the present study. Fifth, the prognosis of patients having AIH with acute hepatitis cannot be determined from this survey, because the subjects were newly diagnosed from 2014 to 2017. Development of internationally uniform diagnostic methods and future surveys of acute AIH will address these limitations.

In conclusion, AIH with acute hepatitis in Japan was shown to have an increasing incidence and changing characteristics. The present nationwide survey suggests the need for new strategies for the accurate diagnosis and elucidation of the mechanisms in acute AIH.

## Methods

### Participants

A total of 138 hospitals (not including pediatric centers) that had hepatology specialists were asked to complete questionnaires about patients who were newly diagnosed with AIH from 2014 to 2017. Of the 138 hospitals, 54 returned the questionnaires from 923 AIH patients. After excluding patients who had been diagnosed with AIH before 2014 and did not satisfy the revised^26^ or simplified criteria^27^ of AIH, a total of 796 patients were enrolled in this study. Of the 1687 patients in the previous nationwide survey in 2015, 1,410 who satisfied the revised or simplified criteria of AIH were also included in this study. The Ethics Committee of Fukushima Medical University (Fukushima Medical University protocol number: 29182) approved this study protocol, which waived the need for written, informed consent because this was an observational study. Instead of written, informed consent, information about this study was released, and participants were given the right to opt out. This survey was conducted in accordance with the Declaration of Helsinki.

## Questionnaire

The questionnaire included items about age at diagnosis; sex; medical and family history; alcohol and history of medication associated with the development of AIH; laboratory and liver histological findings; diagnosis based on the revised and simplified diagnostic scores, as proposed by the International AIH Study Group (IAIHG)^26,27^; diagnosis and severity grading based on the Japanese guidelines^22^; clinical symptoms; imaging findings; presence of other autoimmune or malignant diseases; treatments; and outcomes. The autoantibodies were tested by the methods used in each hospital, such as indirect IF or enzyme-linked immunosorbent assays. Severity grading was classified into mild, moderate, and severe based on a combination of clinical signs (hepatic encephalopathy, reduction or disappearance of hepatic dullness), clinical laboratory tests (AST/ALT > 200 U/L, bilirubin > 5 mg/dL, prothrombin time < 60%), and imaging tests (hepatic atrophy, heterogeneous liver parenchyma pattern) (Supplemental Table 1). Steroid pulse therapy was defined as high-dose i.v. corticosteroids (> 125 mg methylprednisolone/body/day). Similar to a previous survey^5^, in addition to liver histological diagnoses such as acute hepatitis, chronic hepatitis, and cirrhosis, the present survey graded basic histology as interface hepatitis; portal inflammation; plasma cell infiltration; lobular necrosis or inflammation (0, absent; 1, mild; or 2, moderate to severe); fibrosis (0, absent; 1, mild; 2, moderate; 3, severe; or 4, cirrhosis); bile duct injury; hepatocyte rosette formation; centrilobular necrosis; emperipolesis; and fatty change (0, absent; 1, present). Acute hepatitis was defined based on histological diagnosis by the pathologists in each hospital. Biochemical remission was defined as normalization of transaminase (< 30 U/L) and IgG (< 1,700 mg/dL) levels.

### Statistical analysis

The data are presented as medians for continuous variables and as percentages for categorical variables. Differences between the two groups (2018 survey and 2015 survey) were analyzed using the Mann–Whitney *U*-test for continuous variables and the χ^2^ test or Fisher’s exact test for categorical variables. To account for possible bias among the participating hospitals and clinics in the comparison of histological diagnoses, only the 28 facilities that completed both the 2015 and 2018 surveys were analyzed. Statistical analyses were performed with SPSS ver. 25.0 (SPSS Inc., Chicago, IL). A *p* value of < 0.05 was considered significant.

## Supplementary information

Supplementary Information

## Data Availability

The authors do not have permission to share data.

## References

[1] Abe M (2011). Autoimmune hepatitis study group-subgroup of the intractable hepato-biliary disease study Group in Japan. Present status of autoimmune hepatitis in Japan: a nationwide survey. J. Gastroenterol..

[2] Takahashi A (2017). Autoimmune hepatitis in Japan: trends in a nationwide survey. J. Gastroenterol..

[3] Joshita S (2018). Clinical features of autoimmune hepatitis with acute presentation: a Japanese nationwide survey. J. Gastroenterol..

[4] Fujiwara K (2018). Long-term observation of acute-onset autoimmune hepatitis presenting clinically and radiologically as acute hepatitis. Hepatol. Int..

[5] Harada K, Hiep NC, Ohira H (2017). Challenges and difficulties in pathological diagnosis of autoimmune hepatitis. Hepatol. Res..

[6] Nguyen Canh H (2017). Acute presentation of autoimmune hepatitis: a multicentre study with detailed histological evaluation in a large cohort of patients. J. Clin. Pathol..

[7] Dohmen K, Tanaka H, Haruno M, Aishima S (2017). Immunoserological and histological differences between autoimmune hepatitis with acute presentation and chronic autoimmune hepatitis. Hepatol. Res..

[8] Ohira H, Abe K, Takahashi A, Watanabe H (2015). Autoimmune hepatitis: recent advances in the pathogenesis and new diagnostic guidelines in Japan. Intern. Med..

[9] Yamamoto K (2013). Prognosis of autoimmune hepatitis showing acute presentation. Hepatol Res..

[10] Abe K (2012). Centrilobular necrosis in acute presentation of Japanese patients with type 1 autoimmune hepatitis. World J. Hepatol..

[11] Onji M. The Autoimmune Hepatitis Group. Proposal of autoimmune hepatitis presenting with acute hepatitis, severe hepatitis and acute liver failure. Hepatol Res. 41, 497 (2011). 10.1111/j.1872-034X.2011.00810.x 21554505

[12] Fujiwara K, Yasui S, Yokosuka O (2011). Efforts for making the diagnosis of acute onset autoimmune hepatitis. Hepatology.

[13] Takahashi H, Zeniya M (2011). Acute presentation of autoimmune hepatitis: Does it exist? A published work review. Hepatol. Res..

[14] Miyake Y (2010). Autoimmune hepatitis with acute presentation in Japan. Dig. Liver Dis..

[15] Fujiwara K, Fukuda Y, Yokosuka O (2008). Precise histological evaluation of liver biopsy specimen is indispensable for diagnosis and treatment of acute-onset autoimmune hepatitis. J. Gastroenterol..

[16] Abe M (2007). Clinicopathologic features of the severe form of acute type 1 autoimmune hepatitis. Clin. Gastroenterol. Hepatol..

[17] Okano N (2003). Clinicopathological features of acute-onset autoimmune hepatitis. Hepatol. Res..

[18] Abe M (2001). Clinical characteristics of autoimmune hepatitis with histological features of acute hepatitis. Hepatol. Res..

[19] Monna T, Kuroki T, Yamamoto S (1985). Autoimmune hepatitis: the present status in Japan. Gastroenterol. Jpn..

[20] Onji M (1993). Present status of autoimmune hepatitis in Japan. Gastroenterol. Jpn..

[21] Toda G (1997). Present status of autoimmune hepatitis in Japan-correlating the characteristics with international criteria in an area with a high rate of HCV infection. J. Hepatol..

[22] Onji M, Zeniya M, Yamamoto K, Tsubouchi H (2014). Autoimmune hepatitis: Diagnosis and treatment guide in Japan, 2013. Hepatol. Res..

[23] Katanoda K (2016). JACR Monograph Supplement No 2.

[24] Tanaka A (2009). Increase trend in the prevalence and male-to-female ratio of primary biliary cholangitis, autoimmune hepatitis, and primary sclerosing cholangitis in Japan. Hepatol. Res..

[25] European Association for the Study of the Liver (2015). EASL clinical practice guidelines: autoimmune hepatitis. J. Hepatol..

[26] Alzare R (1999). International Autoimmune Hepatitis Group Report: review of criteria for diagnosis of autoimmune hepatitis. J. Hepatol..

[27] Hennes EM (2008). Simplified criteria for the diagnosis of autoimmune hepatitis. Hepatology.

