# Supplementary material for: Increasing incidence of acute autoimmune hepatitis: a nationwide survey in Japan
Source: Sci Rep. 2020 Aug 28;10:14250. doi: 10.1038/s41598-020-71296-0 (PMC7455563; doi:10.1038/s41598-020-71296-0)
Supplement: Supplementary file 1 — Supplementary Information [file 41598_2020_71296_MOESM1_ESM.docx]

**Increasing incidence of acute autoimmune hepatitis: A nationwide survey in Japan**

Atsushi Takahashi^1^, Hiromasa Ohira^1^, Kazumichi Abe^1^, Mikio Zeniya^2^, Masanori Abe^3^, Teruko Arinaga-Hino^4^, Takuji Torimura^4^, Kaname Yoshizawa^5^, Akinobu Takaki^6^, Jong-Hon Kang^7^, Yoshiyuki Suzuki^8^, Nobuhiro Nakamoto^9^, Ayano Inui^10^, Atsushi Tanaka^11^, Hajime Takikawa^12^

^1^Department of Gastroenterology, Fukushima Medical University School of Medicine, 1 Hikarigaoka, Fukushima 960-1295 Japan, ^2^Sanno Medical Center, International University of Health and Welfare, 8-10-16 Akasaka, Minato-ku, Tokyo 107-0052, Japan, ^3^Department of Gastroenterology and Metabology, Ehime University Graduate School of Medicine, Shitsukawa, To-on, Ehime 791-0295, Japan, ^4^Department of Medicine, Kurume University School of Medicine, 67 Asahi-machi, Kurume-shi, Fukuoka 830-0011, Japan, ^5^Department of Gastroenterology, National Hospital Organization, Shinshu Ueda Medical Center, 1-27-21 Midorigaoka, Ueda-City, Nagano 386-8610, Japan, ^6^Department of Gastroenterology and Hepatology, Okayama University Graduate School of Medicine, Dentistry, and Pharmaceutical Sciences, 2-5-1 Shikata-cho, Kita-ku, Okayama-city 700-8558, Japan, ^7^Center for Gastroenterology, Teine Keijinkai Hospital, 1-12 Maeda, Teine-ku, Sapporo 006-8555, Japan, ^8^Department of Hepatology, Toranomon Hospital, Toranomon 2-2-2, Minato-ku, Tokyo 105-8470, Japan, ^9^Department of Internal Medicine, Keio University School of Medicine, 35 Shinanomachi, Shinjuku-ku, Tokyo 160-8582, Japan, ^10^Department of Pediatric Hepatology and Gastroenterology, Saiseikai Yokohamashi Tobu Hospital, 3-6-1 Shimosueyoshi, Tsurumi-ku, Yokohama-City, Kanagawa 230-0012, Japan, ^11^Department of Medicine, Teikyo University School of Medicine, 2-11-1, Kaga, Itabashi-ku, Tokyo 173-8605, Japan, ^12^Faculty of Medical Technology, Teikyo University, 2-11-1, Kaga, Itabashi-ku, Tokyo 173-8605, Japan

Supplemental Table 1. Severity of diagnosis and treatment guide in Japan, 2013 [22]

| Clinical signs | Clinical laboratory tests | Imaging test |
| --- | --- | --- |
| ①Hepatic encephalopathy | ①AST/ALT of more than 200 U/L | ①Hepatic atrophy |
| ②Reduction or disappearance of hepatic dullness | ②Bilirubin of more than 5 mg/dL | ②Heterogeneous liver parenchyma pattern |
|  | ③Prothrombin time of less than 60% |  |
| Severe: should fulfill at least one of these three findings: | | |
| 1 Clinical signs: ① or ② | | |
| 2 Clinical laboratory tests: both ① and ③, or both ② and ③ | | |
| 3 Imaging test: ① or ② | | |
| Moderate: should fulfill these findings: | | |
| Clinical laboratory tests: one of the criteria (①, ② or ③) or both ① and ②, without clinical signs (neither ① nor ②), and imaging tests (neither ① nor ② ) | | |
| Mild: none of the above criteria are observed. | | |

ALT, alanine aminotransferase; AST, aspartate aminotransferase

Supplemental Table 2. Histologic diagnoses in 2015 and 2018 in the 28 facilities completed both the 2015 and 2018 surveys.

| Histologic diagnosis | 2015 (516) | 2018 (459) | *P* |
| --- | --- | --- | --- |
| Acute hepatitis | 13.8% (71) | 23.7% (109) | <0.001 |
| Chronic hepatitis | 77.7% (401) | 67.3% (309) | <0.001 |
| Liver cirrhosis | 8.5% (44) | 8.9% (41) | 0.912 |

Values are given as the percentage (number).
